# Supplementary material for: Spatial structure, chemotaxis and quorum sensing shape bacterial biomass accumulation in complex porous media
Source: Nat Commun. 2024 Jan 2;15:191. doi: 10.1038/s41467-023-44267-y (PMC10761857; doi:10.1038/s41467-023-44267-y)
Supplement: Supplementary file 1 — Supplementary information [file 41467_2023_44267_MOESM1_ESM.pdf]

Supplementary information for Spatial structure, chemotaxis and quorum sensing shape bacterial biomass accumulation in complex porous media

David Scheidweiler[1], Ankur Deep Bordoloi[1], Wenqiao Jiao[1], Vladimir Sentchilo[2], Monica Bollani[3], Audam Chhun[2], Philipp Engel[2] and Pietro de Anna[1]

[1] Institute of Earth Sciences, University of Lausanne, CH-1015 Lausanne, Switzerland

[2]Department of Fundamental Microbiology, University of Lausanne, CH-1015 Lausanne, Switzerland

[3] IFN-CNR, L-NESS, Via Anzani 42, 22100, Como, Italy

[§] Corresponding author: pietro.deanna@unil.ch and david.scheidweiler@unil.ch

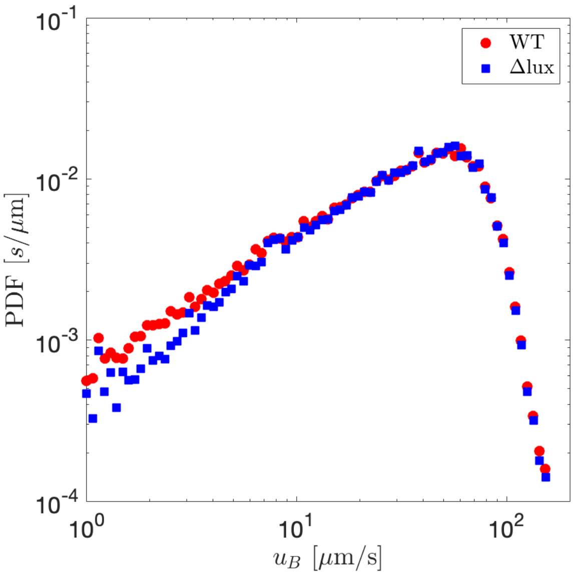

Figure S1. Distribution of the swimming velocities of WT (red) and Δ*luxS* **(**blue**)** derived from the statistical analysis of particle tracking performed in absence of flow (one way t-test, $p > 0.1$).

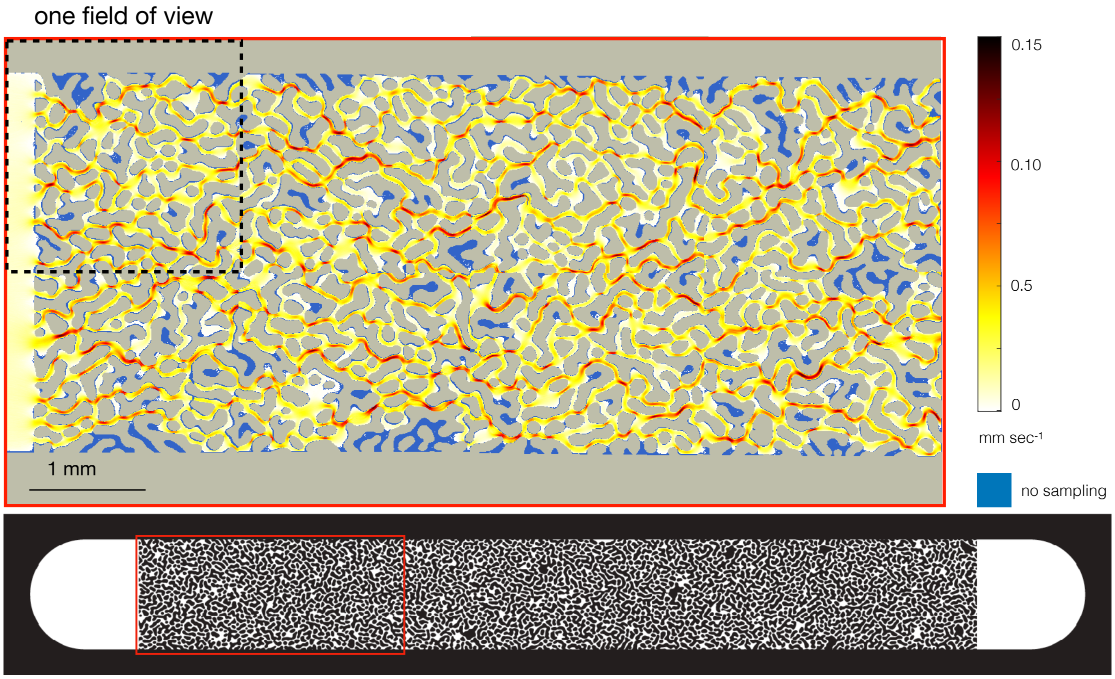

Figure S2. Top. Velocity field measured with Particle Image Velocimetry (as in Bordoloi et al. Nat. Comm. 2022), by stitching together 8 camera fields of view (the dashed line represents the top left one). The PIV experiment is conducted continuously injecting at Q=0.1 uL/s fluorescent microspheres of 0.5 microns in diameter (Thermofisher, FLUOROMAX B05) visualized with a Nikon DAPI filter cube. The colormap represents the measured velocity in mm/s, while the blue zones are no-sampling regions where the local flow is so low that no particles entered the region.

To assess the velocity field in such regions we decided to use numerical simulations. Bottom. The red rectangle identifies the portion of the whole microfluidics for which the PIV has been performed.

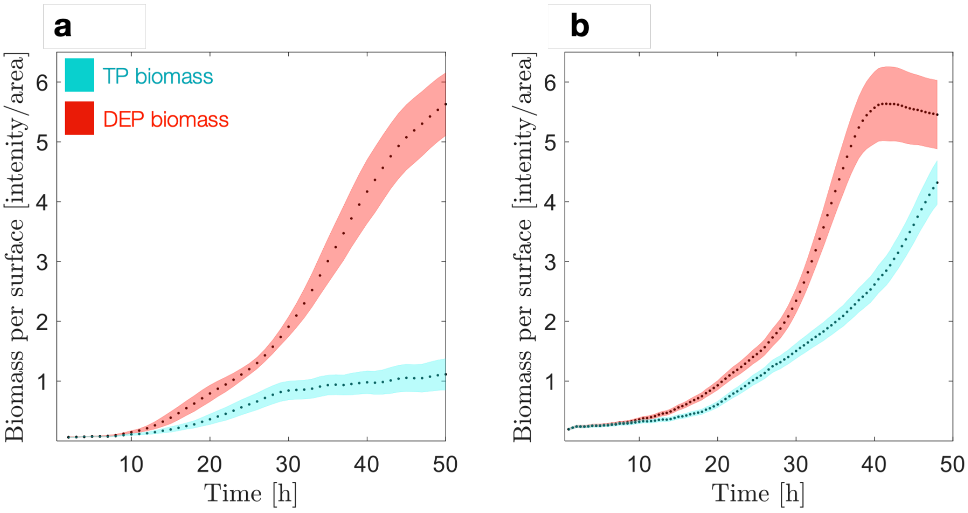

Figure S3. Impact of glucose concentration on *E. coli* WT biomass partitioning between TPs and DEPs. Comparison of biomass concentration per pore type, for (A) experiments performed at glucose concentration of 5 mM, and (B) experiments performed at glucose concentration of 50 mM. Dots represent averages of independent experiments (n=3); shaded areas represent standard deviation.

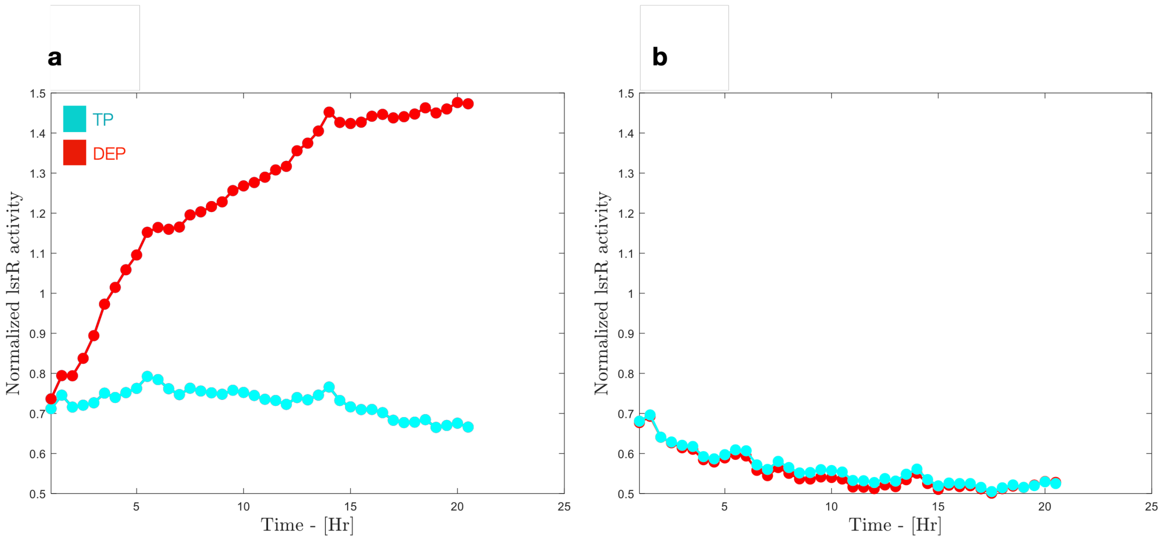

Figure S4. Impact of glucose concentration on *E. coli lsrR* activity. Comparison of normalized *lsrR* activity per pore type, red for dead-end pores, cyan for transmitting pores. (A) Experiments performed in Lysogeny Broth diluted in distilled water 10 times (LB 0.1) without glucose, (repeated measures ANOVA, $p < 0.0001$). (B) Experiments performed at with LB 0.1 and glucose 5 mM (repeated measures ANOVA, $p > 0.1$).

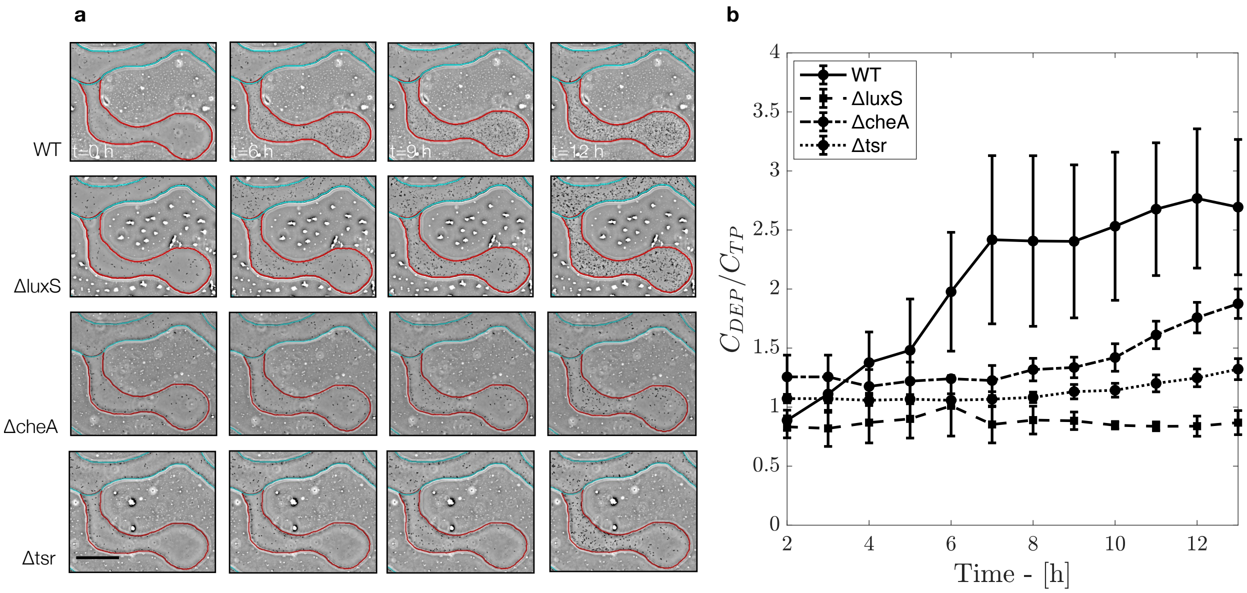

Figure S5. Transport mediated accumulation of *E. coli* at early times (0-12 hours). (A) Images at different times representing bacterial cells abundance in a single DEP, highlighted in red, and the nearest TP, highlighted in cyan, for *E. coli* WT, *E. coli ΔluxS*, *E. coli ΔcheA* and *E. coli Δtsr* (from top to bottom). Scale bar 50 µm. (B) Retention curves representing the ratio between the concentration of bacteria in the dead-end pores (C_{DEP}) and their concentration in the transmitting pores (C_{TP}) at different times, for all the four strains mentioned above. Dots represent averages of independent experiments (n=3); bars represent standard deviation.

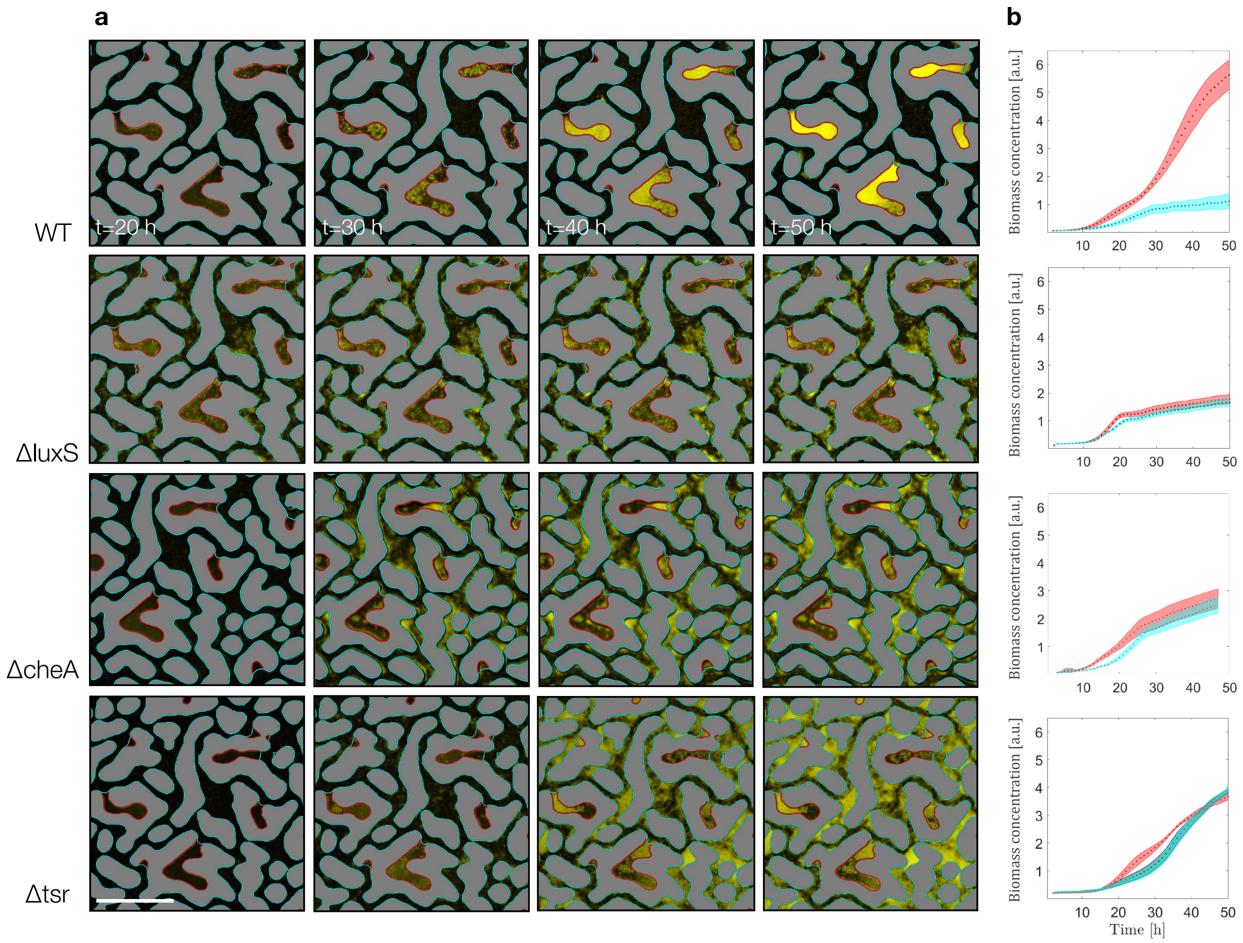

Figure S6. Macroscopic biomass accumulation, *E. coli* WT, *E. coli ΔluxS*, *E. coli ΔcheA* and *E. coli Δtsr* (from top to bottom). (A) Overview of microfluidic chip depicting biased biomass accumulation through the microfluidic geometry at 20, 30, 40 ad 50 hours since nutrient injection. Scale bar 200 µm. (B) Temporal evolution of the biomass concentration in the TP (cyan), in the DEP (red). Dots represent averages of independent experiments (n=3); shaded areas represent standard deviation.

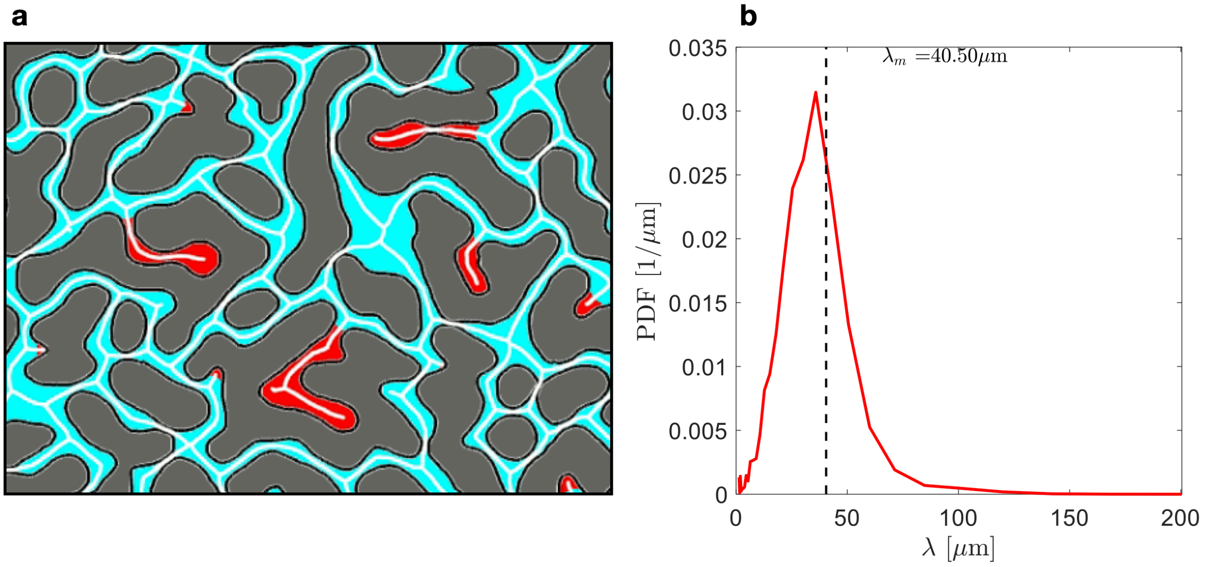

Figure S7. Pore throat characterization. (A) Representation of the binarized map where TP are colored in cyan, DEP in red, and skeleton in white. (B) Distribution of pore throats measured along the skeleton of the system, dashed line indicates average pore throat size, λ_m = 40 µm.

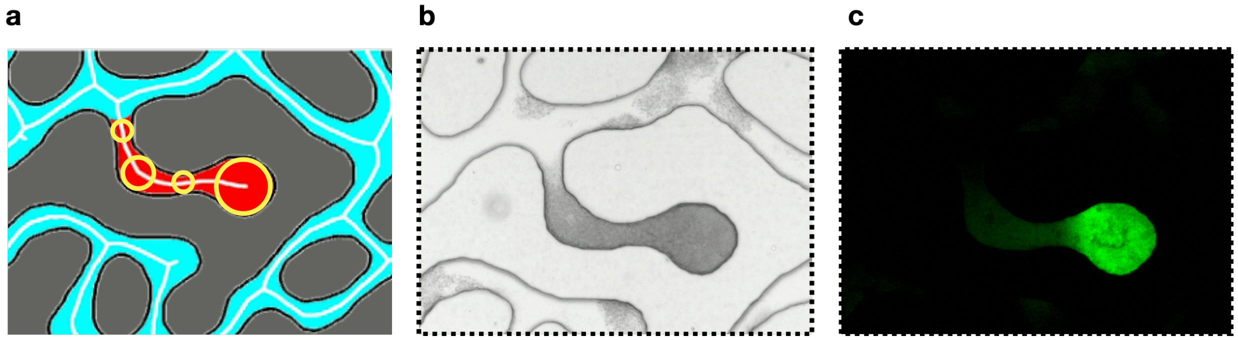

Figure S8. Pore scale measurement of biomass and *lsrR* activity. (A) Image intensities have been averaged over a disk of radius *r* centered along the skeleton of each individual DEP structure, every 10 pixels. In figure have been plotted only 4 representative circles along the skeleton. This allowed to quantify (B) the biomass accumulation from the bright field images, and in the same way to estimate (C) the *lsrR* activity from the reporter fluorescence signal.

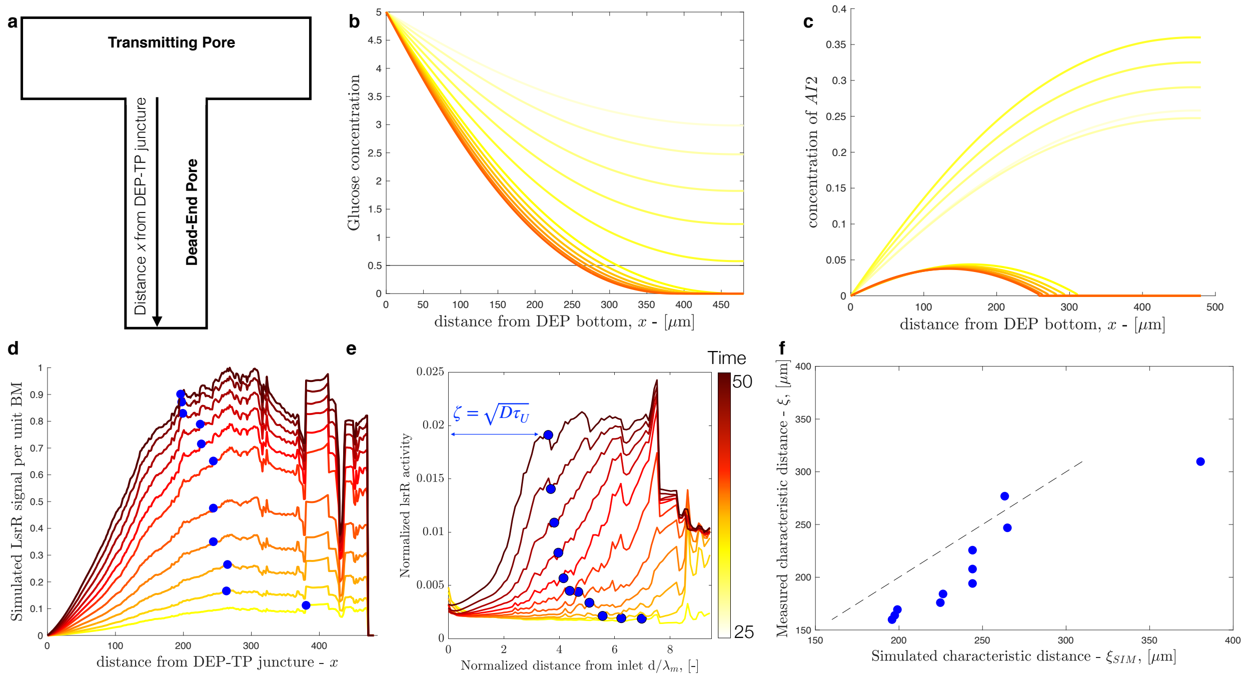

Figure S9. Single DEP, one dimensional diffusive-reactive system. (a) schematic representation of a single DEP: x = 0 represent the DEP-TP juncture. (b) Numerical solution of the glucose concentration (in mM) that diffuses and is consumed by the resident biomass (measured), at different times, from light to dark - increasing time. (c) Numerical solution of the AI-2 that is produced by cells: while it diffuses, it is consumed by cells where glucose is below a the arbitrary threshold of 0.5 mM (10% of the initial Glucose concentration). (d) Estimated *lsrR* signal, based on simulated AI-2 and measured biomass (BM), dots represent the 80% maximum signal. e) Measured *lsrR* signal as function of distance from inlet, dots represent diffusive length scale $\sqrt{D\tau_U}$. (f) Report the characteristic distance ζ measured (blue dots in e) and simulated (blue dots in d).
