# Supplementary material for: Spatial structure, chemotaxis and quorum sensing shape bacterial biomass accumulation in complex porous media
Source: Nat Commun. 2024 Jan 2;15:191. doi: 10.1038/s41467-023-44267-y (PMC10761857; doi:10.1038/s41467-023-44267-y)
Supplement: Supplementary file 3 — Description of Additional Supplementary Files [file 41467_2023_44267_MOESM3_ESM.pdf]

Description of Additional Supplementary Files:

Supplementary Movie 1: Biomass accumulation for Wild Type. Time lapse (1h time interval) of biomass dynamics over the entire time of the experiment. Biomass signal, computed from bright field images, is highlighted in yellow over black background. Grains of the porous geometry are highlighted in gray, DEPs boundary in red, TPs in cyan.

Supplementary Movie 2: Biomass accumulation for ΔluxS. Time lapse (1h time interval) of biomass dynamics over the entire time of the experiment. Biomass signal, computed from bright field images, is highlighted in yellow over black background. Grains of the porous geometry are highlighted in gray, DEPs boundary in red, TPs in cyan.

Supplementary Movie 3: Cells DEP vs TP colonization at early times for Wild Type. Phase contrast images representing cell colonization for the first 19 h (1 h time interval). DEPs boundary are highlighted in red, TPs in cyan.

Supplementary Movie 4: Cells DEP vs TP colonization at early times for ΔluxS. Phase contrast images representing cell colonization for the first 19 h (1 h time interval). DEPs boundary are highlighted in red, TPs in cyan.
